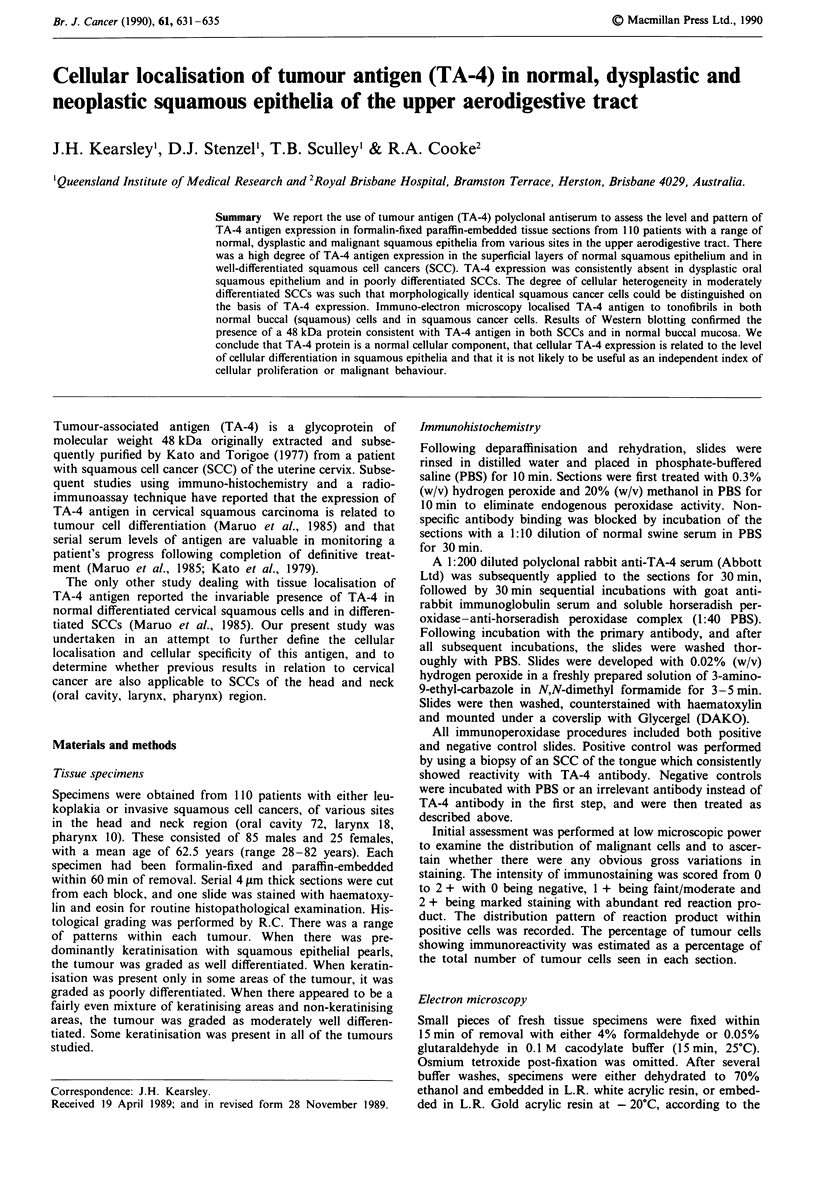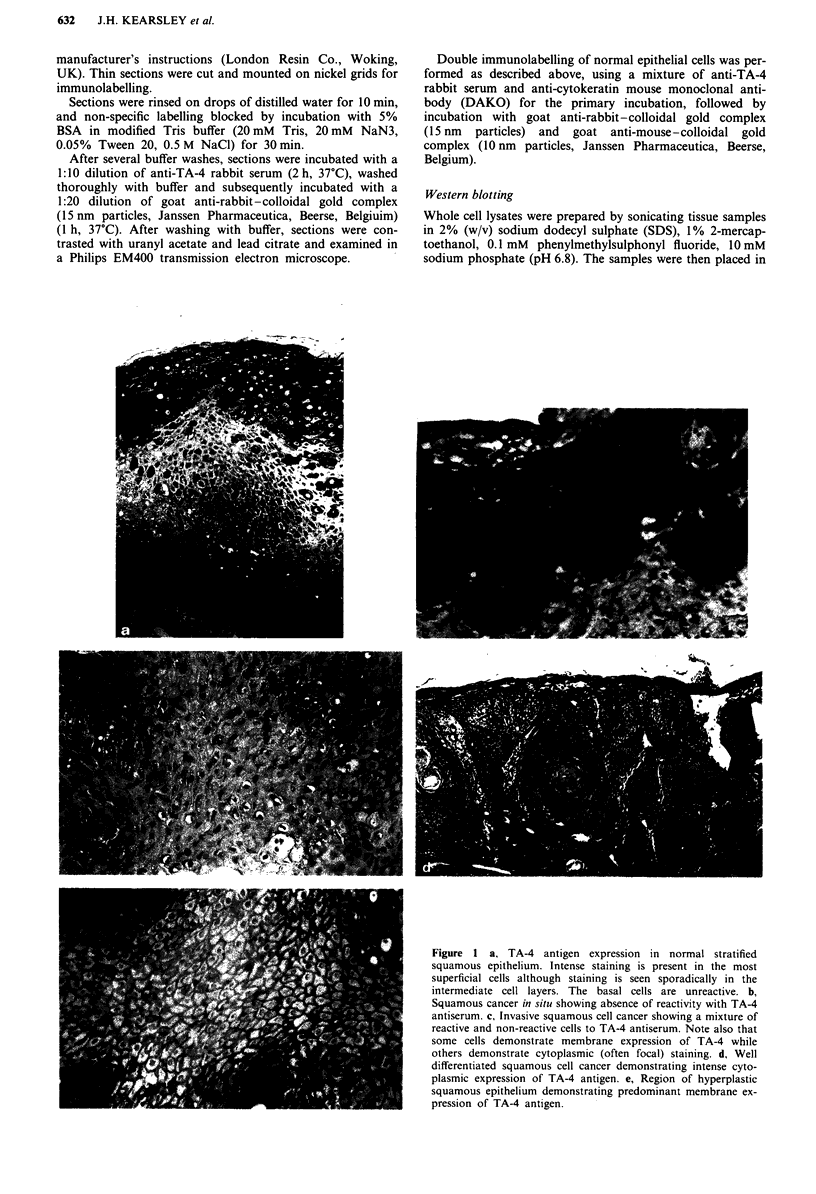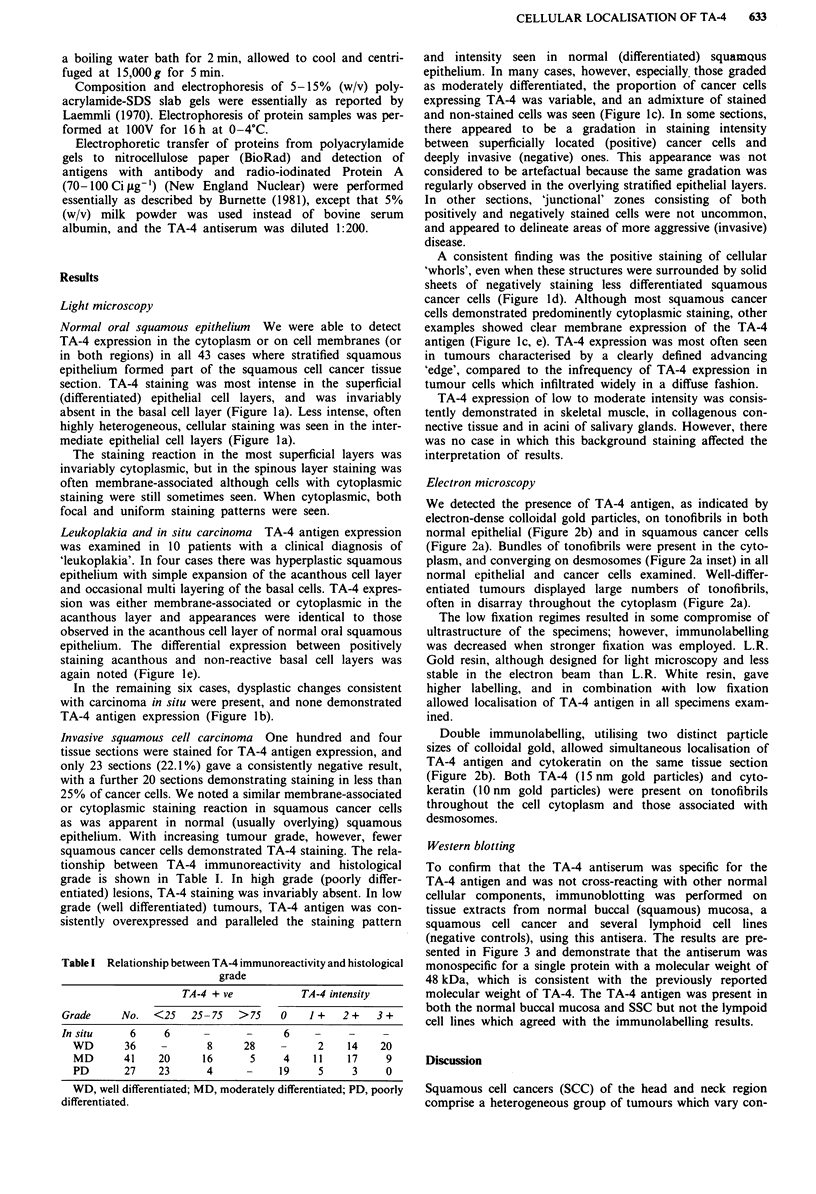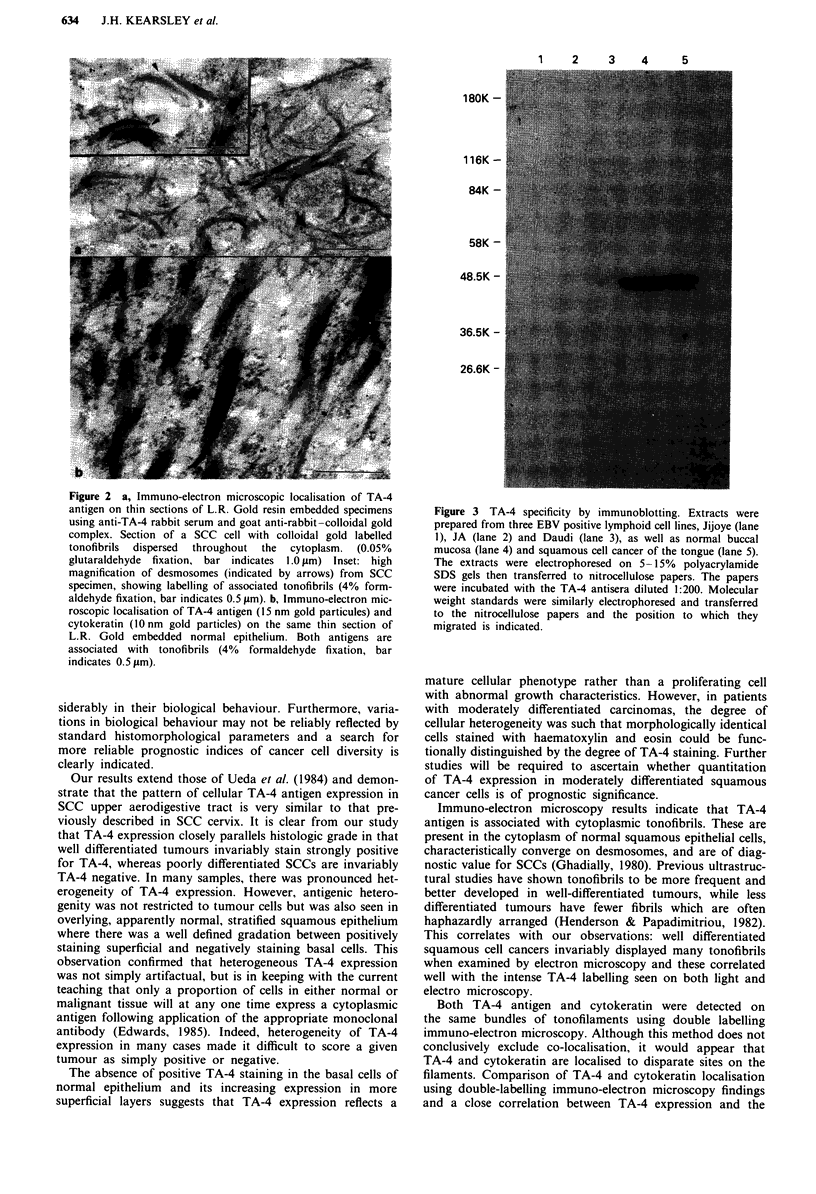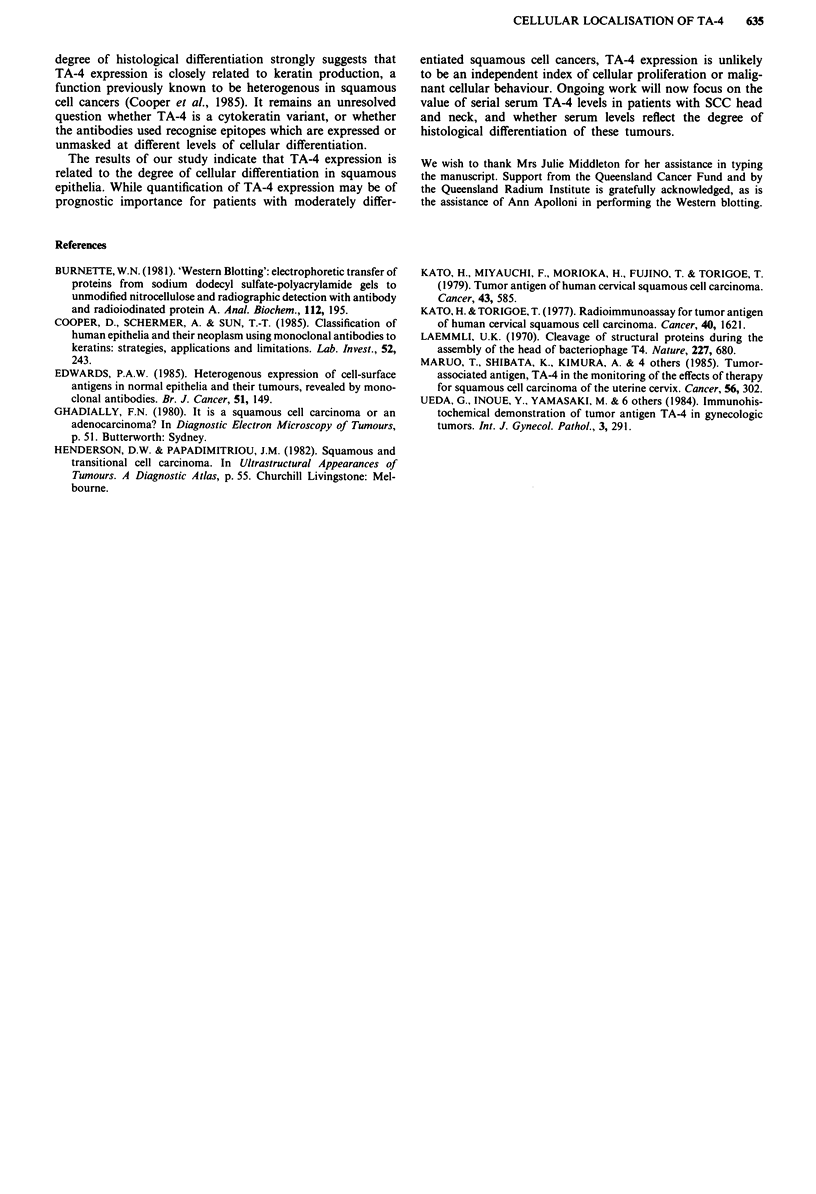# Cellular localisation of tumour antigen (TA-4) in normal, dysplastic and neoplastic squamous epithelia of the upper aerodigestive tract.

**DOI:** 10.1038/bjc.1990.140

**Published:** 1990-04

**Authors:** J. H. Kearsley, D. J. Stenzel, T. B. Sculley, R. A. Cooke

**Affiliations:** Queensland Institute of Medical Research, Australia.

## Abstract

**Images:**


*Br. J. Cancer* (1990), **61**, 631–635 © Macmillan Press Ltd., 1990

### Cellular localisation of tumour antigen (TA-4) in normal, dysplastic and neoplastic squamous epithelia of the upper aerodigestive tract

J.H. Kearsley[1], D.J. Stenzel[1], T.B. Sculley[1] & R.A. Cooke[2]

[1]*Queensland Institute of Medical Research and* [2]*Royal Brisbane Hospital, Bramston Terrace, Herston, Brisbane 4029, Australia.*

**Summary** We report the use of tumour antigen (TA-4) polyclonal antiserum to assess the level and pattern of TA-4 antigen expression in formalin-fixed paraffin-embedded tissue sections from 110 patients with a range of normal, dysplastic and malignant squamous epithelia from various sites in the upper aerodigestive tract. There was a high degree of TA-4 antigen expression in the superficial layers of normal squamous epithelium and in well-differentiated squamous cell cancers (SCC). TA-4 expression was consistently absent in dysplastic oral squamous epithelium and in poorly differentiated SCCs. The degree of cellular heterogeneity in moderately differentiated SCCs was such that morphologically identical squamous cancer cells could be distinguished on the basis of TA-4 expression. Immuno-electron microscopy localised TA-4 antigen to tonofibrils in both normal buccal (squamous) cells and in squamous cancer cells. Results of Western blotting confirmed the presence of a 48 kDa protein consistent with TA-4 antigen in both SCCs and in normal buccal mucosa. We conclude that TA-4 protein is a normal cellular component, that cellular TA-4 expression is related to the level of cellular differentiation in squamous epithelia and that it is not likely to be useful as an independent index of cellular proliferation or malignant behaviour.

Tumour-associated antigen (TA-4) is a glycoprotein of molecular weight 48 kDa originally extracted and subsequently purified by Kato and Torigoe (1977) from a patient with squamous cell cancer (SCC) of the uterine cervix. Subsequent studies using immuno-histochemistry and a radioimmunoassay technique have reported that the expression of TA-4 antigen in cervical squamous carcinoma is related to tumour cell differentiation (Maruo *et al.*, 1985) and that serial serum levels of antigen are valuable in monitoring a patient's progress following completion of definitive treatment (Maruo *et al.*, 1985; Kato *et al.*, 1979).

The only other study dealing with tissue localisation of TA-4 antigen reported the invariable presence of TA-4 in normal differentiated cervical squamous cells and in differentiated SCCs (Maruo *et al.*, 1985). Our present study was undertaken in an attempt to further define the cellular localisation and cellular specificity of this antigen, and to determine whether previous results in relation to cervical cancer are also applicable to SCCs of the head and neck (oral cavity, larynx, pharynx) region.

### Materials and methods

### *Tissue specimens*

Specimens were obtained from 110 patients with either leukoplakia or invasive squamous cell cancers, of various sites in the head and neck region (oral cavity 72, larynx 18, pharynx 10). These consisted of 85 males and 25 females, with a mean age of 62.5 years (range 28–82 years). Each specimen had been formalin-fixed and paraffin-embedded within 60 min of removal. Serial 4 μm thick sections were cut from each block, and one slide was stained with haematoxylin and eosin for routine histopathological examination. Histological grading was performed by R.C. There was a range of patterns within each tumour. When there was predominantly keratinisation with squamous epithelial pearls, the tumour was graded as well differentiated. When keratinisation was present only in some areas of the tumour, it was graded as poorly differentiated. When there appeared to be a fairly even mixture of keratinising areas and non-keratinising areas, the tumour was graded as moderately well differentiated. Some keratinisation was present in all of the tumours studied.

Correspondence: J.H. Kearsley.
Received 19 April 1989; and in revised form 28 November 1989.

### *Immunohistochemistry*

Following deparaffinisation and rehydration, slides were rinsed in distilled water and placed in phosphate-buffered saline (PBS) for 10 min. Sections were first treated with 0.3% (w/v) hydrogen peroxide and 20% (w/v) methanol in PBS for 10 min to eliminate endogenous peroxidase activity. Non-specific antibody binding was blocked by incubation of the sections with a 1:10 dilution of normal swine serum in PBS for 30 min.

A 1:200 diluted polyclonal rabbit anti-TA-4 serum (Abbott Ltd) was subsequently applied to the sections for 30 min, followed by 30 min sequential incubations with goat anti-rabbit immunoglobulin serum and soluble horseradish peroxidase–anti-horseradish peroxidase complex (1:40 PBS). Following incubation with the primary antibody, and after all subsequent incubations, the slides were washed thoroughly with PBS. Slides were developed with 0.02% (w/v) hydrogen peroxide in a freshly prepared solution of 3-amino-9-ethyl-carbazole in *N,N*-dimethyl formamide for 3–5 min. Slides were then washed, counterstained with haematoxylin and mounted under a coverslip with Glycergel (DAKO).

All immunoperoxidase procedures included both positive and negative control slides. Positive control was performed by using a biopsy of an SCC of the tongue which consistently showed reactivity with TA-4 antibody. Negative controls were incubated with PBS or an irrelevant antibody instead of TA-4 antibody in the first step, and were then treated as described above.

Initial assessment was performed at low microscopic power to examine the distribution of malignant cells and to ascertain whether there were any obvious gross variations in staining. The intensity of immunostaining was scored from 0 to 2 + with 0 being negative, 1 + being faint/moderate and 2 + being marked staining with abundant red reaction product. The distribution pattern of reaction product within positive cells was recorded. The percentage of tumour cells showing immunoreactivity was estimated as a percentage of the total number of tumour cells seen in each section.

### *Electron microscopy*

Small pieces of fresh tissue specimens were fixed within 15 min of removal with either 4% formaldehyde or 0.05% glutaraldehyde in 0.1 M cacodylate buffer (15 min, 25°C). Osmium tetroxide post-fixation was omitted. After several buffer washes, specimens were either dehydrated to 70% ethanol and embedded in L.R. white acrylic resin, or embedded in L.R. Gold acrylic resin at – 20°C, according to the manufacturer's instructions (London Resin Co., Woking, UK). Thin sections were cut and mounted on nickel grids for immunolabelling.

Sections were rinsed on drops of distilled water for 10 min, and non-specific labelling blocked by incubation with 5% BSA in modified Tris buffer (20 mM Tris, 20 mM NaN3, 0.05% Tween 20, 0.5 M NaCl) for 30 min.

After several buffer washes, sections were incubated with a 1:10 dilution of anti-TA-4 rabbit serum (2 h, 37°C), washed thoroughly with buffer and subsequently incubated with a 1:20 dilution of goat anti-rabbit–colloidal gold complex (15 nm particles, Janssen Pharmaceutica, Beerse, Belgiuim) (1 h, 37°C). After washing with buffer, sections were contrasted with uranyl acetate and lead citrate and examined in a Philips EM400 transmission electron microscope.

Double immunolabelling of normal epithelial cells was performed as described above, using a mixture of anti-TA-4 rabbit serum and anti-cytokeratin mouse monoclonal antibody (DAKO) for the primary incubation, followed by incubation with goat anti-rabbit–colloidal gold complex (15 nm particles) and goat anti-mouse–colloidal gold complex (10 nm particles, Janssen Pharmaceutica, Beerse, Belgium).

### *Western blotting*

Whole cell lysates were prepared by sonicating tissue samples in 2% (w/v) sodium dodecyl sulphate (SDS), 1% 2-mercaptoethanol, 0.1 mM phenylmethylsulphonyl fluoride, 10 mM sodium phosphate (pH 6.8). The samples were then placed in

**Figure 1** **a**, TA-4 antigen expression in normal stratified squamous epithelium. Intense staining is present in the most superficial cells although staining is seen sporadically in the intermediate cell layers. The basal cells are unreactive. **b**, Squamous cancer *in situ* showing absence of reactivity with TA-4 antiserum. **c**, Invasive squamous cell cancer showing a mixture of reactive and non-reactive cells to TA-4 antiserum. Note also that some cells demonstrate membrane expression of TA-4 while others demonstrate cytoplasmic (often focal) staining. **d**, Well differentiated squamous cell cancer demonstrating intense cytoplasmic expression of TA-4 antigen. **e**, Region of hyperplastic squamous epithelium demonstrating predominant membrane expression of TA-4 antigen.

a boiling water bath for 2 min, allowed to cool and centrifuged at 15,000 *g* for 5 min.

Composition and electrophoresis of 5–15% (w/v) polyacrylamide-SDS slab gels were essentially as reported by Laemmli (1970). Electrophoresis of protein samples was performed at 100V for 16 h at 0–4°C.

Electrophoretic transfer of proteins from polyacrylamide gels to nitrocellulose paper (BioRad) and detection of antigens with antibody and radio-iodinated Protein A (70–100 Ci $\mu g^{-1}$) (New England Nuclear) were performed essentially as described by Burnette (1981), except that 5% (w/v) milk powder was used instead of bovine serum albumin, and the TA-4 antiserum was diluted 1:200.

### Results

### *Light microscopy*

*Normal oral squamous epithelium* We were able to detect TA-4 expression in the cytoplasm or on cell membranes (or in both regions) in all 43 cases where stratified squamous epithelium formed part of the squamous cell cancer tissue section. TA-4 staining was most intense in the superficial (differentiated) epithelial cell layers, and was invariably absent in the basal cell layer (Figure 1a). Less intense, often highly heterogeneous, cellular staining was seen in the intermediate epithelial cell layers (Figure 1a).

The staining reaction in the most superficial layers was invariably cytoplasmic, but in the spinous layer staining was often membrane-associated although cells with cytoplasmic staining were still sometimes seen. When cytoplasmic, both focal and uniform staining patterns were seen.

*Leukoplakia and in situ carcinoma* TA-4 antigen expression was examined in 10 patients with a clinical diagnosis of 'leukoplakia'. In four cases there was hyperplastic squamous epithelium with simple expansion of the acanthous cell layer and occasional multi layering of the basal cells. TA-4 expression was either membrane-associated or cytoplasmic in the acanthous layer and appearances were identical to those observed in the acanthous cell layer of normal oral squamous epithelium. The differential expression between positively staining acanthous and non-reactive basal cell layers was again noted (Figure 1e).

In the remaining six cases, dysplastic changes consistent with carcinoma *in situ* were present, and none demonstrated TA-4 antigen expression (Figure 1b).

*Invasive squamous cell carcinoma* One hundred and four tissue sections were stained for TA-4 antigen expression, and only 23 sections (22.1%) gave a consistently negative result, with a further 20 sections demonstrating staining in less than 25% of cancer cells. We noted a similar membrane-associated or cytoplasmic staining reaction in squamous cancer cells as was apparent in normal (usually overlying) squamous epithelium. With increasing tumour grade, however, fewer squamous cancer cells demonstrated TA-4 staining. The relationship between TA-4 immunoreactivity and histological grade is shown in Table I. In high grade (poorly differentiated) lesions, TA-4 staining was invariably absent. In low grade (well differentiated) tumours, TA-4 antigen was consistently overexpressed and paralleled the staining pattern and intensity seen in normal (differentiated) squamous epithelium. In many cases, however, especially those graded as moderately differentiated, the proportion of cancer cells expressing TA-4 was variable, and an admixture of stained and non-stained cells was seen (Figure 1c). In some sections, there appeared to be a gradation in staining intensity between superficially located (positive) cancer cells and deeply invasive (negative) ones. This appearance was not considered to be artefactual because the same gradation was regularly observed in the overlying stratified epithelial layers. In other sections, 'junctional' zones consisting of both positively and negatively stained cells were not uncommon, and appeared to delineate areas of more aggressive (invasive) disease.

**Table I** Relationship between TA-4 immunoreactivity and histological grade

| | | *TA-4 + ve* | | | *TA-4 intensity* | | | |
|---|---|---|---|---|---|---|---|---|
| *Grade* | *No.* | *<25* | *25–75* | *>75* | *0* | *1+* | *2+* | *3+* |
| *In situ* | 6 | 6 | – | – | 6 | – | – | – |
| WD | 36 | – | 8 | 28 | – | 2 | 14 | 20 |
| MD | 41 | 20 | 16 | 5 | 4 | 11 | 17 | 9 |
| PD | 27 | 23 | 4 | – | 19 | 5 | 3 | 0 |

WD, well differentiated; MD, moderately differentiated; PD, poorly differentiated.

A consistent finding was the positive staining of cellular 'whorls', even when these structures were surrounded by solid sheets of negatively staining less differentiated squamous cancer cells (Figure 1d). Although most squamous cancer cells demonstrated predominently cytoplasmic staining, other examples showed clear membrane expression of the TA-4 antigen (Figure 1c, e). TA-4 expression was most often seen in tumours characterised by a clearly defined advancing 'edge', compared to the infrequency of TA-4 expression in tumour cells which infiltrated widely in a diffuse fashion.

TA-4 expression of low to moderate intensity was consistently demonstrated in skeletal muscle, in collagenous connective tissue and in acini of salivary glands. However, there was no case in which this background staining affected the interpretation of results.

### *Electron microscopy*

We detected the presence of TA-4 antigen, as indicated by electron-dense colloidal gold particles, on tonofibrils in both normal epithelial (Figure 2b) and in squamous cancer cells (Figure 2a). Bundles of tonofibrils were present in the cytoplasm, and converging on desmosomes (Figure 2a inset) in all normal epithelial and cancer cells examined. Well-differentiated tumours displayed large numbers of tonofibrils, often in disarray throughout the cytoplasm (Figure 2a).

The low fixation regimes resulted in some compromise of ultrastructure of the specimens; however, immunolabelling was decreased when stronger fixation was employed. L.R. Gold resin, although designed for light microscopy and less stable in the electron beam than L.R. White resin, gave higher labelling, and in combination with low fixation allowed localisation of TA-4 antigen in all specimens examined.

Double immunolabelling, utilising two distinct particle sizes of colloidal gold, allowed simultaneous localisation of TA-4 antigen and cytokeratin on the same tissue section (Figure 2b). Both TA-4 (15 nm gold particles) and cytokeratin (10 nm gold particles) were present on tonofibrils throughout the cell cytoplasm and those associated with desmosomes.

### *Western blotting*

To confirm that the TA-4 antiserum was specific for the TA-4 antigen and was not cross-reacting with other normal cellular components, immunoblotting was performed on tissue extracts from normal buccal (squamous) mucosa, a squamous cell cancer and several lymphoid cell lines (negative controls), using this antisera. The results are presented in Figure 3 and demonstrate that the antiserum was monospecific for a single protein with a molecular weight of 48 kDa, which is consistent with the previously reported molecular weight of TA-4. The TA-4 antigen was present in both the normal buccal mucosa and SSC but not the lympoid cell lines which agreed with the immunolabelling results.

### Discussion

Squamous cell cancers (SCC) of the head and neck region comprise a heterogeneous group of tumours which vary con-

**Figure 2** **a**, Immuno-electron microscopic localisation of TA-4 antigen on thin sections of L.R. Gold resin embedded specimens using anti-TA-4 rabbit serum and goat anti-rabbit–colloidal gold complex. Section of a SCC cell with colloidal gold labelled tonofibrils dispersed throughout the cytoplasm. (0.05% glutaraldehyde fixation, bar indicates 1.0 μm) Inset: high magnification of desmosomes (indicated by arrows) from SCC specimen, showing labelling of associated tonofibrils (4% formaldehyde fixation, bar indicates 0.5 μm). **b**, Immuno-electron microscopic localisation of TA-4 antigen (15 nm gold particules) and cytokeratin (10 nm gold particles) on the same thin section of L.R. Gold embedded normal epithelium. Both antigens are associated with tonofibrils (4% formaldehyde fixation, bar indicates 0.5 μm).

**Figure 3** TA-4 specificity by immunoblotting. Extracts were prepared from three EBV positive lymphoid cell lines, Jijoye (lane 1), JA (lane 2) and Daudi (lane 3), as well as normal buccal mucosa (lane 4) and squamous cell cancer of the tongue (lane 5). The extracts were electrophoresed on 5–15% polyacrylamide SDS gels then transferred to nitrocellulose papers. The papers were incubated with the TA-4 antisera diluted 1:200. Molecular weight standards were similarly electrophoresed and transferred to the nitrocellulose papers and the position to which they migrated is indicated.

siderably in their biological behaviour. Furthermore, variations in biological behaviour may not be reliably reflected by standard histomorphological parameters and a search for more reliable prognostic indices of cancer cell diversity is clearly indicated.

Our results extend those of Ueda *et al.* (1984) and demonstrate that the pattern of cellular TA-4 antigen expression in SCC upper aerodigestive tract is very similar to that previously described in SCC cervix. It is clear from our study that TA-4 expression closely parallels histologic grade in that well differentiated tumours invariably stain strongly positive for TA-4, whereas poorly differentiated SCCs are invariably TA-4 negative. In many samples, there was pronounced heterogeneity of TA-4 expression. However, antigenic heterogenity was not restricted to tumour cells but was also seen in overlying, apparently normal, stratified squamous epithelium where there was a well defined gradation between positively staining superficial and negatively staining basal cells. This observation confirmed that heterogeneous TA-4 expression was not simply artifactual, but is in keeping with the current teaching that only a proportion of cells in either normal or malignant tissue will at any one time express a cytoplasmic antigen following application of the appropriate monoclonal antibody (Edwards, 1985). Indeed, heterogeneity of TA-4 expression in many cases made it difficult to score a given tumour as simply positive or negative.

The absence of positive TA-4 staining in the basal cells of normal epithelium and its increasing expression in more superficial layers suggests that TA-4 expression reflects a mature cellular phenotype rather than a proliferating cell with abnormal growth characteristics. However, in patients with moderately differentiated carcinomas, the degree of cellular heterogeneity was such that morphologically identical cells stained with haematoxylin and eosin could be functionally distinguished by the degree of TA-4 staining. Further studies will be required to ascertain whether quantitation of TA-4 expression in moderately differentiated squamous cancer cells is of prognostic significance.

Immuno-electron microscopy results indicate that TA-4 antigen is associated with cytoplasmic tonofibrils. These are present in the cytoplasm of normal squamous epithelial cells, characteristically converge on desmosomes, and are of diagnostic value for SCCs (Ghadially, 1980). Previous ultrastructural studies have shown tonofibrils to be more frequent and better developed in well-differentiated tumours, while less differentiated tumours have fewer fibrils which are often haphazardly arranged (Henderson & Papadimitriou, 1982). This correlates with our observations: well differentiated squamous cell cancers invariably displayed many tonofibrils when examined by electron microscopy and these correlated well with the intense TA-4 labelling seen on both light and electro microscopy.

Both TA-4 antigen and cytokeratin were detected on the same bundles of tonofilaments using double labelling immuno-electron microscopy. Although this method does not conclusively exclude co-localisation, it would appear that TA-4 and cytokeratin are localised to disparate sites on the filaments. Comparison of TA-4 and cytokeratin localisation using double-labelling immuno-electron microscopy findings and a close correlation between TA-4 expression and the degree of histological differentiation strongly suggests that TA-4 expression is closely related to keratin production, a function previously known to be heterogenous in squamous cell cancers (Cooper *et al.*, 1985). It remains an unresolved question whether TA-4 is a cytokeratin variant, or whether the antibodies used recognise epitopes which are expressed or unmasked at different levels of cellular differentiation.

The results of our study indicate that TA-4 expression is related to the degree of cellular differentiation in squamous epithelia. While quantification of TA-4 expression may be of prognostic importance for patients with moderately differentiated squamous cell cancers, TA-4 expression is unlikely to be an independent index of cellular proliferation or malignant cellular behaviour. Ongoing work will now focus on the value of serial serum TA-4 levels in patients with SCC head and neck, and whether serum levels reflect the degree of histological differentiation of these tumours.

We wish to thank Mrs Julie Middleton for her assistance in typing the manuscript. Support from the Queensland Cancer Fund and by the Queensland Radium Institute is gratefully acknowledged, as is the assistance of Ann Apolloni in performing the Western blotting.

### References

BURNETTE, W.N. (1981). 'Western Blotting': electrophoretic transfer of proteins from sodium dodecyl sulfate-polyacrylamide gels to unmodified nitrocellulose and radiographic detection with antibody and radioiodinated protein A. *Anal. Biochem.*, **112**, 195.

COOPER, D., SCHERMER, A. & SUN, T.-T. (1985). Classification of human epithelia and their neoplasm using monoclonal antibodies to keratins: strategies, applications and limitations. *Lab. Invest.*, **52**, 243.

EDWARDS, P.A.W. (1985). Heterogenous expression of cell-surface antigens in normal epithelia and their tumours, revealed by monoclonal antibodies. *Br. J. Cancer*, **51**, 149.

GHADIALLY, F.N. (1980). It is a squamous cell carcinoma or an adenocarcinoma? In *Diagnostic Electron Microscopy of Tumours*, p. 51. Butterworth: Sydney.

HENDERSON, D.W. & PAPADIMITRIOU, J.M. (1982). Squamous and transitional cell carcinoma. In *Ultrastructural Appearances of Tumours. A Diagnostic Atlas*, p. 55. Churchill Livingstone: Melbourne.

KATO, H., MIYAUCHI, F., MORIOKA, H., FUJINO, T. & TORIGOE, T. (1979). Tumor antigen of human cervical squamous cell carcinoma. *Cancer*, **43**, 585.

KATO, H. & TORIGOE, T. (1977). Radioimmunoassay for tumor antigen of human cervical squamous cell carcinoma. *Cancer*, **40**, 1621.

LAEMMLI, U.K. (1970). Cleavage of structural proteins during the assembly of the head of bacteriophage T4. *Nature*, **227**, 680.

MARUO, T., SHIBATA, K., KIMURA, A. & 4 others (1985). Tumor-associated antigen, TA-4 in the monitoring of the effects of therapy for squamous cell carcinoma of the uterine cervix. *Cancer*, **56**, 302.

UEDA, G., INOUE, Y., YAMASAKI, M. & 6 others (1984). Immunohistochemical demonstration of tumor antigen TA-4 in gynecologic tumors. *Int. J. Gynecol. Pathol.*, **3**, 291.